# Circuit-Level Transient Simulation Study of Recovery Characteristics in a Parallel-Resonator-Assisted GaN SBD Limiter

**DOI:** 10.3390/mi17070790

**Published:** 2026-06-28

**Authors:** Rikang Zhao, Yu Zhang, Xiansong Tian, Xiangguan Tan, Shaohang Xu, Haitao Zhang, Da Chen, Juinjei Liou

**Affiliations:** 1 College of Electronic and Information Engineering, Shandong University of Science and Technology, Qingdao 266590, China; xiansongtianwy@163.com (X.T.); tanxiangguan@sdust.edu.cn (X.T.); chenda@sdust.edu.cn (D.C.); juin.liou@hotmail.com (J.L.); 2 Beijing Institute of Measurement and Test, Beijing 100086,China; zhangyustudy@126.com; 3 Inspur Computer Technology Co., Ltd., Jinan 250101, China; 4 Ningbo Daxin Semiconductor Co., Ltd., Ningbo 315400, China; tony_zhang@daxin-semi.com

**Keywords:** fast recovery, GaN, limiter, SBD, transient simulation

## Abstract

In this work, fast-recovery characteristics of high-power GaN Schottky barrier diode (SBD) limiters are investigated through a parallel-resonator-assisted topology and a circuit-level transient simulation method for recovery-process analysis. By introducing a parallel resonant unit, composed of the diode junction capacitance and short-circuited stubs, into a conventional shunt limiter topology, the high-frequency passband characteristics of the traditional shunt topology under large-size GaN SBD conditions are improved, and the transient response after high-power excitation is reshaped, thereby shortening the recovery time from the large-signal limiting state to the small-signal steady-state operation. Based on the ADS platform, a combined excitation scheme using a high-power pulse signal and a low-power continuous-wave signal is adopted to perform circuit-level transient simulation of the limiter recovery process. Simulation results show that, under a 50-W input power condition, the recovery time is reduced from 16.3 ns to 11.2 ns after introducing the parallel resonant unit in the C-band. These results indicate that circuit topology significantly affects the recovery behavior of high-power GaN SBD limiters, and the proposed approach provides a useful reference for circuit-level design and transient analysis of fast-recovery microwave limiters.

## 1. Introduction

Microwave limiters are critical front-end control devices in modern RF receivers, where the continuous increase in operating frequency and transmission power levels places higher demands on both the typical RF performance and specific performance of limiters. Typical RF performance such as insertion loss, return loss, threshold, and IP3 must meet requirements for high linearity, low loss, and adjustable threshold [1]. However, optimizing these parameters alone is insufficient to address the challenges of complex operating environments. Limiters must also exhibit fast recovery time [2], near-instantaneous response capability [3], and robustness under high-power conditions [4,5].

In practical applications, omitting the limiter requires relying on the high-power handling capability of GaN LNAs to protect the receiver from damage. However, this approach significantly increases system DC power consumption and costs [6,7]. As a result, PIN diode limiters have become the most commonly implemented solution. While this method is suitable for many legacy designs, its narrow bandwidth, long recovery time [8], and high spike leakage [9] limit its application in advanced transceiver systems. State-of-the-art systems often demand limiters that simultaneously achieve high power capacity, high linearity, and instantaneous recovery capabilities, posing significant challenges for conventional PIN diode limiters [10].

With the continuous advancement of GaN unipolar device technology, GaN Schottky barrier diode (SBD) technology has gradually matured. Benefiting from the majority-carrier transport mechanism, GaN SBDs theoretically do not suffer from the minority-carrier storage effect observed in conventional PIN diodes, thus exhibiting near-zero reverse recovery characteristics. This provides a promising solution for overcoming the tradeoff between power-handling capability and recovery time in traditional microwave limiters. Meanwhile, the intrinsic material properties of GaN, including high breakdown electric field, high electron mobility, and excellent thermal stability further make GaN SBDs highly attractive for high-power microwave limiter applications [11].

However, in practical high-power GaN SBD limiter designs, larger device dimensions are generally required to improve RF current-handling capability and reduce on-resistance, which inevitably introduce increased junction capacitance and energy-storage effects. Under high-power pulse excitation, the accumulation and release of stored charge within the device still lead to noticeable recovery delay, preventing GaN SBD limiters from achieving the theoretically expected near-zero recovery characteristics. In addition, the increased junction capacitance also results in reduced cutoff frequency and degraded high-frequency performance. Therefore, relying solely on the intrinsic fast carrier transport mechanism of the device is insufficient to further resolve the tradeoff between recovery performance and high-frequency operation under high-power conditions [12].

Current research on microwave limiters mainly focuses on improving power capacity, operating bandwidth, and linearity, whereas studies on recovery time are still largely limited to experimental characterization and phenomenological observations [13,14,15]. Particularly for high-power GaN SBD limiters, the recovery process involves complex nonlinear dynamic behaviors including charge accumulation, transient energy release, and topology-dependent energy-storage paths, yet systematic transient simulation and mechanism analysis of recovery characteristics remain insufficiently explored. Furthermore, limited attention has been paid to the influence of circuit topology on recovery behavior, making recovery-time optimization still highly dependent on device fabrication and material properties rather than circuit-level design methodologies.

In this work, a resonator-assisted high-power GaN SBD limiter topology is investigated based on equivalent-circuit simulation to analyze both recovery and linearity characteristics. By introducing a parallel resonant unit formed by short-circuited stubs and the junction capacitance of the SBDs into a conventional shunt limiter topology, the proposed structure not only improves the high-frequency passband characteristics but also establishes an additional transient charge-discharge path to enhance recovery performance under high-power excitation. Based on the ADS transient simulation platform, a recovery-time simulation methodology combining high-power pulse excitation with low-power continuous-wave signals is further developed to investigate the recovery processes of different limiter topologies. In addition, the linearity and high-power characteristics of the limiter, including OIP3, harmonic suppression, and power-handling capability, are systematically analyzed through simulation. The simulation results demonstrate that the proposed resonator-assisted topology effectively improves the recovery characteristics of high-power GaN SBD limiters while maintaining favorable high-frequency and high-linearity performance.

## 2. GaN Device Prototyping and Equivalent-Circuit Model

### 2.1. Scalable Model for GaN SBD 

**Figure 1:**
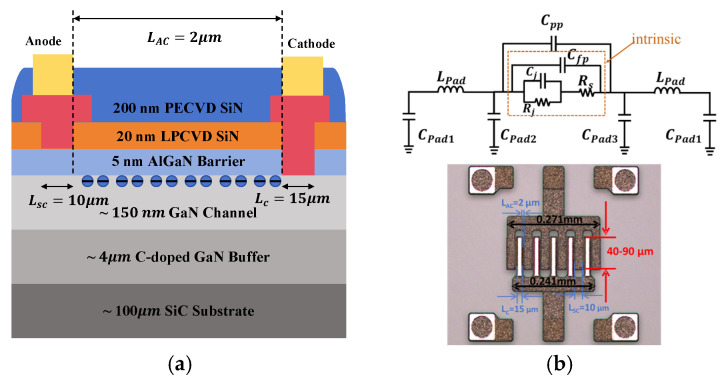
(**a**) Cross-sectional schematic of recess-free thin-barrier lateral heterojunction AlGaN/GaN SBD. (**b**) GaN SBD device layout structure and equivalent circuit model.

**Figure 2:**
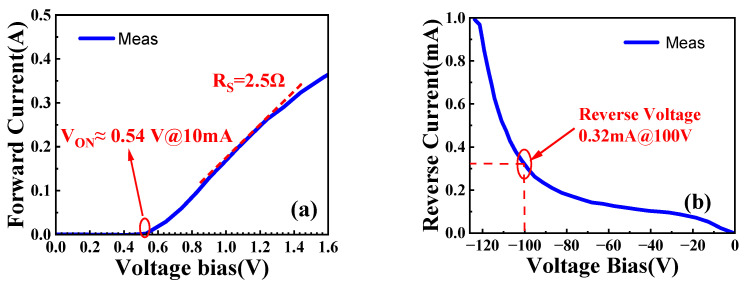
The characteristics of GaN SBD. (**a**) Forward I-V characteristic curve with linear axis. (**b**) Reverse characteristic curve.

In our previous work [16], a GaN Schottky barrier diode (SBD) with a low turn-on voltage (V_on_ < 0.6 V) was developed based on a recess-free thin-barrier process. Figure 1a illustrates the device layout and cross-sectional structure. The geometrical parameters of the device are as follows: the anode-to-cathode spacing (L_AC_) is 2 μm, the cathode length (L_SC_) is 10 μm, and the anode diameter (L_C_) is 15 μm. Figure 1b shows the optical micrograph of the fabricated AlGaN/GaN SBD with a total anode width (W_a_) of 500 μm. The device exhibits excellent reverse-blocking capability, with a leakage current as low as 0.32 mA under a reverse bias of 100 V, as shown in Figure 2. 

For the practical application of GaN SBDs in high-frequency circuits, it is necessary to establish a broadband equivalent circuit model suitable for circuit-level simulation. The accuracy of such a model mainly depends on the reliable extraction of intrinsic S-parameters and associated parasitic parameters over a wide frequency range.

Figure 1b shows the small-signal equivalent circuit of a lateral AlGaN/GaN heterojunction SBD. Owing to the relatively large device size, parasitic capacitances C_pad_ and parasitic inductances L_pad_ are introduced by the anode and cathode metallization, respectively. In addition, the coupling effect between the anode and cathode metallization gives rise to an inter-electrode coupling capacitance C_pp_. As shown in the equivalent circuit, the model parameters consist of two parts: parasitic parameters and intrinsic parameters. The parasitic parameters include the capacitances of three different pad structures, denoted as C_pad1_, C_pad2_, and C_pad3_, the coupling capacitance C_pp_ between two pads, and the feedline inductance L_pad_. The intrinsic parameters mainly include the junction capacitance C_j_, the capacitance introduced by the field-plate structure C_fp_, the junction resistance R_j_, and the series resistance R_s_.

**Figure 3:**
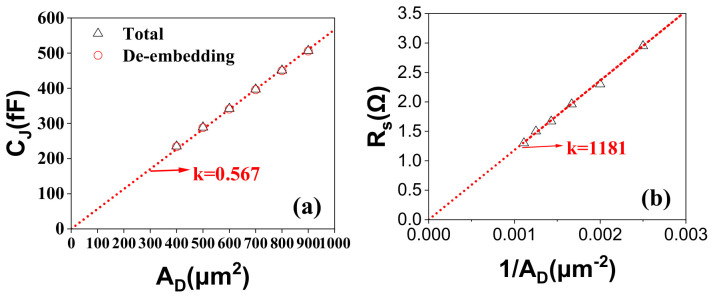
(**a**) The relationship between the intrinsic junction capacitance of an SBD and its effective junction area. (**b**) The relationship between the series resistance of an SBD and its effective junction area.

In general, a large-size device can be regarded as a combination of multiple elementary cells connected in parallel or series; therefore, the scaling rule for the model parameters of a standard elementary cell is of critical importance. The extracted intrinsic junction capacitance and series resistance of six SBDs with different total anode widths are plotted as functions of the effective junction area A_D_, as shown in Figure 3. It can be observed that the intrinsic junction capacitance of the SBD is proportional to A_D_, whereas the series resistance is inversely proportional to A_D_. Accordingly, the scalable modeling rules can be expressed as follows [17]:

(1) $$C_{J}=C_{J}^{0}AD$$

(2) $$Rs=\frac{R_{s}^{0}}{AD}.$$

**Figure 4:**
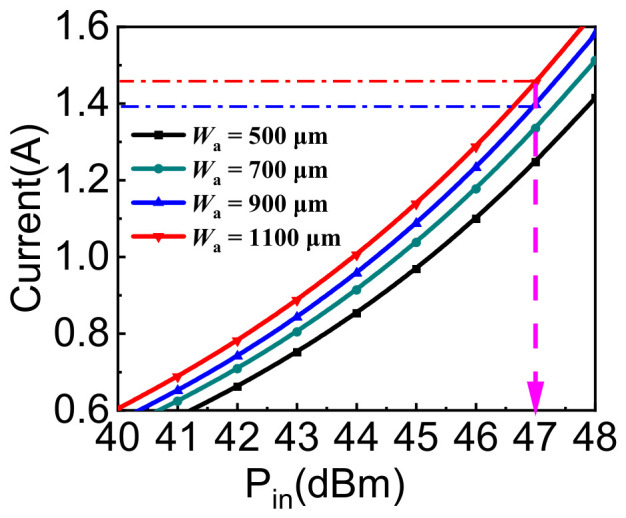
Simulation of the current variation with incident power for single-stage limiters with SBDs of different anode widths *W*_a_.

Here, $C_{j,0}^{0}$ and $R_{s}^{0}$ are referred to as the scaling factors. The extracted model parameters of the SBD with a unit anode width of 1 μm, including both parasitic and intrinsic parameters, are summarized in Table 1. In practical circuit design, device selection can be flexibly optimized by adjusting the total anode width, thereby tuning the current-handling capability of the device. Specifically, reducing the total anode width leads to a smaller junction capacitance at the expense of an increased on-resistance. This design strategy is fundamentally constrained by the tradeoff between the device resistance and capacitance. To evaluate the applicable power-handling capability of the device, RF peak-current simulations were carried out for SBDs with different anode widths W_a_. Figure 4 presents the simulated RF peak current under different input power levels using the proposed model. As the total anode width W_a_ increases, the device is capable of sustaining a higher RF peak current.

### 2.2. GaN SBD Limiter Topology and Equivalent Circuit Model 

Traditional broadband limiters often use a parallel diode topology. To meet the demands for high power capacity, the primary consideration in diode design is typically the device ability to dissipate RF current during forward conduction [15]. This work aims to develop a fast recovery limiter topology suitable for high CW power levels at C-band. When the input power is 50 W, the total peak current flowing through the diode can be approximately calculated as follows [18]:

(3) $$I=\frac{\sqrt{8Z_{0}P_{in}}}{Z_{0}},$$

According to the current division principle of the parallel shunt circuit, the current flowing through the diode is calculated to be 1.4 A. Therefore, when the total anode width is increased to $W_{a}=1100\;\mu m$, the device can withstand the RF current corresponding to an input power below 47 dBm (50 W). Accordingly, an SBD with this anode width can be designed to achieve high power-handling capability.

**Figure 5:**
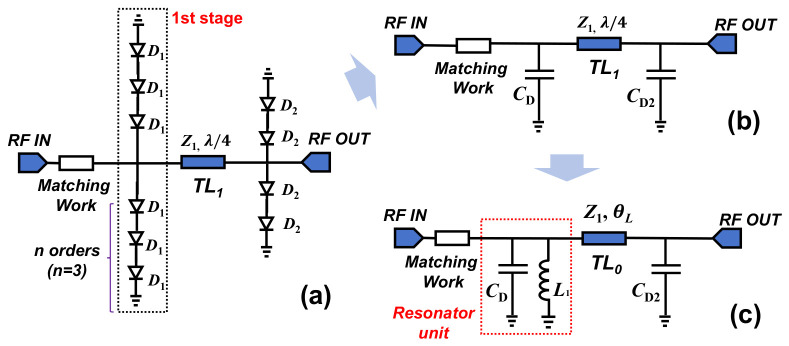
(**a**) Schematic of the traditional parallel diode limiter topology and quarter-wavelength topology. (**b**) Equivalent small-signal topology of the limiter. (**c**) Small-signal equivalent topology of the limiter incorporating a parallel resonant unit of this work.

If a traditional parallel limiter topology is used, as shown in Figure 5a, the cutoff frequency of the designed limiter can be calculated based on [16]:

(4) $$C=\frac{\cos\theta }{w_{0}Z_{0}}=\frac{\cos\theta }{2\pi fZ_{0}},$$

Here, *f* represents the cutoff frequency of the limiter, and the electrical length of the inter-stage transmission line is designated as *λ*/4. From (4), the cutoff frequency of the limiter designed with a device of *W*_a_ = 1100 μm can be calculated to be only 3 GHz, which is not suitable for high frequency range applications. Therefore, to increase the cutoff frequency, a parallel resonator is introduced to transform the low-pass characteristic of the limiter into a band-pass characteristic, thus meeting the required operating frequency range. Due to the introduction of the parallel resonator, the branch structure changes from Figure 5b,c.

**Figure 6:**
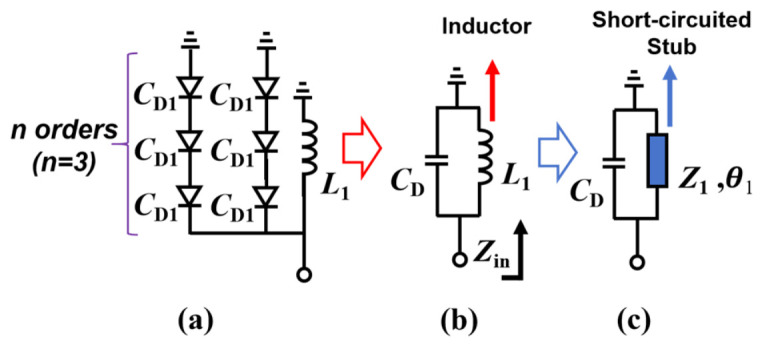
Schematics of the parallel resonator of the first-stage limiter unit: (**a**) Resonator structure. (**b**) Equivalent circuit with lumped components. (**c**) Equivalent circuit after replacing the spiral inductance with an inductive short-circuited stub.

The parallel resonator is a commonly used tuning method in band-pass filters, which can analyze the circuit through a first-stage limiter in Figure 6. By introducing an inductor combined with the equivalent capacitance of the diode C_D_, a resonant unit is formed [19]. The parallel branch reduces the overall branch equivalent capacitance by increasing the number of diodes connected in series. When the orders *n* = 3, the total equivalent capacitance of the diodes in the parallel branch is:

(5) $$C_{D}=2\times \frac{1}{1/C_{D1}+1/C_{D1}+1/C_{D1}}=\frac{2C_{D1}}{3}$$

The required shunt inductance can be calculated from the resonant frequency:

(6) $$L_{1}=\frac{1}{w_{0}^{2}C_{D}}=\frac{3}{2{\left(2\pi f_{0}\right)}^{2}C_{D}}$$

When *C*_D1_ = 0.72 pF and the resonance frequency *f*_0_ is set to 6 GHz, the inductance *L*_1_ can be calculated to be 1.47 nH based on (6). In Figure 6b, to mitigate the impacts of lumped spiral inductors, such as low Q value at high frequency and complex parasitic effects, and to reduce insertion loss without affecting power capability, a short-circuited stub is used to replace the lumped spiral inductor *L*_1_. To ensure the high current-carrying capability of the transmission line, the microstrip width is designed to be 40 μm based on the existing manufacturing process, with the characteristic impedance of *Z*_1_ and an electrical length of *θ*_1_. The empirical conversion formula between the spiral inductor and the corresponding short-circuited stub line can be derived as follows:

(7) $$w_{0}L_{1}=Z_{1}\tan\theta_{1}$$

The electrical length *θ*_1_ of the short-circuit stub can be calculated as:

(8) $$\theta_{1}=\arctan(\frac{3}{4\pi f_{0}C_{D1}Z_{1}})$$

When *θ*_1_ = 36.7°, the short-circuited stub can effectively replace the spiral inductor. Additionally, considering the band-pass characteristics, the 3-dB fractional bandwidth can be calculated as follows:

(9) $$BW=\sqrt{\frac{Z_{1}\tan\theta_{1}}{w_{0}C_{D}}}$$

**Figure 7:**
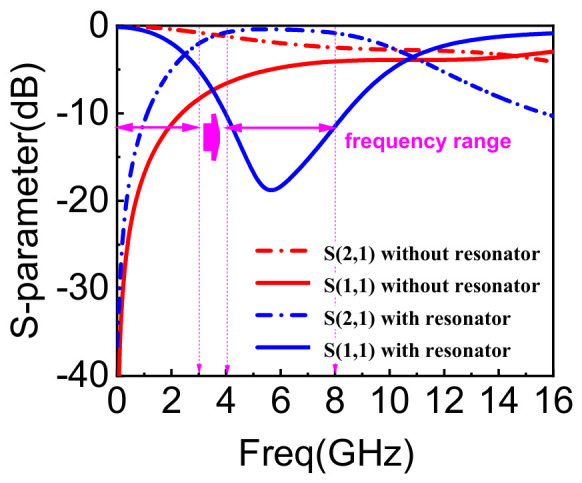
Simulation of the variation in resonant frequency and S-parameter changes of the limiter before and after the introduction of the resonator.

As *θ*_1_ increases, the equivalent inductance *L*_1_ also increases, which leads to a decrease in the resonant frequency of the resonant unit and a corresponding expansion of the 3-dB fractional bandwidth. Therefore, during the design process, the operating frequency range for the C-band was prioritized. Under the condition that the insertion loss is less than 1 dB, both the band-pass characteristics and the area size were considered in collaboration to optimize the short-circuited stub. Ultimately, the electrical length was determined to be *θ*_1_ = 32°, with a physical length of approximately 1815 μm. Figure 7 shows the S-parameter changes of the limiter before and after introducing the inductor. The traditional parallel topology limiter exhibits a low-pass characteristic, with a return loss greater than −4 dB and an insertion loss exceeding 2 dB from DC to 3 GHz. After introducing the parallel resonant unit, the small-signal characteristics of the limiter meet the operation frequency range. 

## 3. Simulation and Performance Analysis of the Limiter MMIC

**Figure 8:**
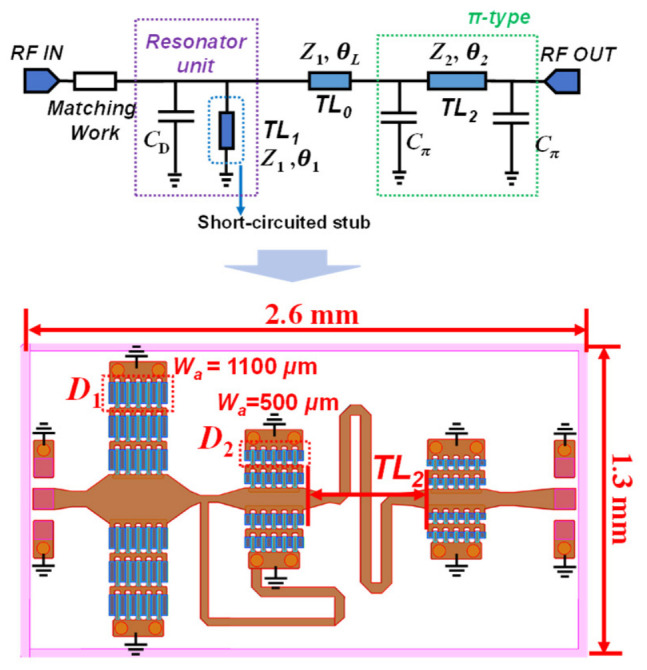
The layout of GaN-SBD MMIC limiter.

To better manage the flat leakage power of the subsequent stages under high-power conditions, the limiter employs a three-stage topology; while the first stage utilizes a parallel resonant topology, the latter two stages both adopt a parallel topology structure, as illustrated in Figure 8. Based on the above design approach, and using the equivalent circuit model of GaN SBD, the inter-stage matching network of the limiter was simulated and optimized using Advanced Design System (ADS). To effectively reduce the area of the limiter MMIC, the inter-stage matching network for the latter stage was replaced with an equivalent π-type instead of a quarter-wavelength topology. Since the diodes in the subsequent stage do not need to handle peak power, a diode with a smaller anode width *W*_a_ = 500 μm was used. Based on the calculation value from (4) and layout EM simulation optimization, the inter-stage electrical length was finally determined to be *θ*_2_ ≈ 45° (1.91 mm) when the limiter cutoff frequency was set to *f* =8 GHz. Table 2 presents the parameters of the circuit using both the traditional quarter-wavelength topology and the improved π-type topology, as well as the final MMIC area. To ensure miniaturization, the short-circuit stub is grounded with the second-stage diodes, and the size of the MMIC is 2.6 mm × 1.3 mm × 0.1 mm.

### 3.1. SBD Limiter MMICs’ Characteristic

**Figure 9:**
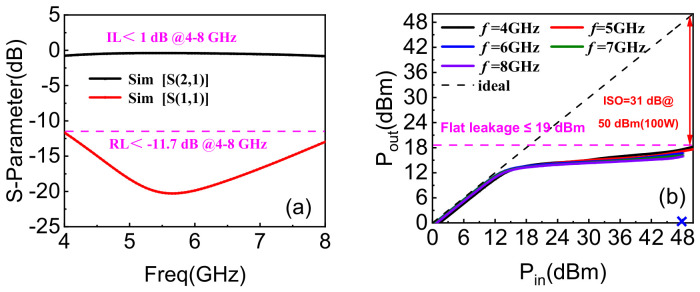
(**a**) Simulated S-parameters of the GaN SBD limiter MMIC. (**b**) Limiting characteristics from 4 GHz to 8 GHz (step by 1 GHz) in CW mode.

The simulation results of the limiter under small signal conditions are shown in Figure 9a. In the range of 4 GHz to 8 GHz, the insertion loss (IL) of the limiter is less than 1.5 dB and the return loss (RL) is better than −11.7 dB. The limiter characteristics at different frequencies are shown in Figure 9b. When the input power exceeds 40 dBm, the flat leakage power decreases with the increase of frequency, which is consistent with the characteristics of the limiter we designed previously. In addition, due to the predefined maximum RF current of the SBDs, the harmonic balance (HB) simulation fails to converge when the input power reaches CW 48 dBm @ 6 GHz. As discussed in Section 2.2, the upper boundary of the adopted large-signal SBD model is defined according to the RF peak-current capability estimated from (3) and the simulation results in Figure 4. For the selected SBD with Wa = 1100 μm, this boundary corresponds to an input power of approximately 48 dBm and an RF peak current of about 1.6 A. When the input power approaches this boundary at 6 GHz under CW excitation, the nonlinear device enters an operating region that has not been calibrated in the equivalent-circuit model. As a result, the HB solver cannot obtain a stable periodic solution. This non-convergence should therefore be regarded as an indication of the model-validity boundary rather than a simple numerical artifact. However, the power capacity of the limiter can still exceed 100 W at lower frequencies. For example, when *f* = 4 GHz with the input power of 50 dBm, the flat leakage is less than 19 dBm, and the isolation can reach 31 dB. 

### 3.2. Recovery Time of the Limiter

**Figure 10:**
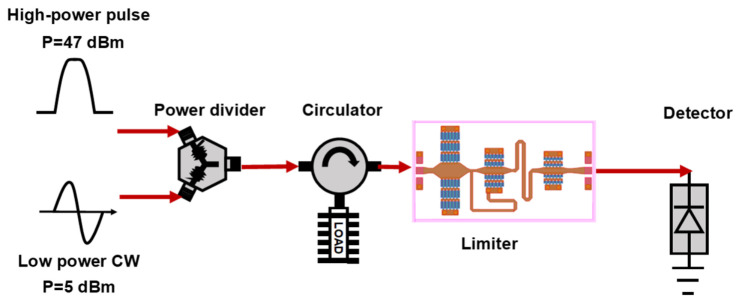
Simulation Schematic for Limiter Recovery Characteristics.

**Figure 11:**
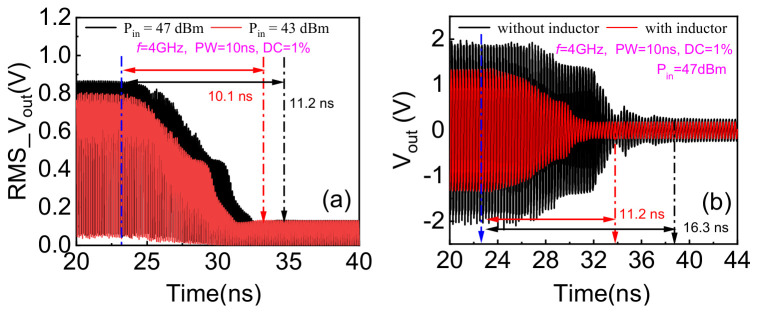
Recovery time of the GaN SBD limiter MMIC: (**a**) Variation of recovery time with incident power, comparing the device anode width (*W*_a_) and with or without the inductor at 4 GHz. (**b**) Impact of inductor on recovery time at 4 GHz.

To investigate the impact of the parallel resonator structure on the recovery characteristics of the limiter, a conventional parallel-type limiter topology with the same devices but without the parallel short-circuited stub was designed as a reference. To compare the recovery performance of the two topologies at 4 GHz, a simulation strategy combining high-power pulses with low-power continuous waves was employed. To ensure phase consistency between the two signals, an ideal power divider as a combiner was introduced at the input for combined injection, as shown in Figure 10. Considering the actual response characteristics of the signal source, the pulse rise/fall time was set to approximately 10 ns, with a pulse width of 10 ns and a duty cycle of 1%. The recovery time is extracted from the end of the falling edge of the high-power pulse to the first time point at which the RMS output voltage returns to and remains within ±5% of this small-signal steady-state reference level. The excitation sources included a 47 dBm pulse signal and a 5 dBm continuous wave. Using the time-domain control module in ADS, the complete recovery process—from the withdrawal of the large signal to the return to small-signal steady state—was simulated, and the RMS value of the output voltage waveform was extracted as the observation indicator. Figure 11a presents the time-domain output characteristics of the proposed fast-recovery limiter under different input power. The results show that as the input power increases, more charge accumulates within the device, leading to a longer recovery time. Under the 50 W condition, the recovery time is approximately 1 ns longer than that under 10 W. Figure 11b shows the effect of the parallel short-circuited stub on the recovery time. The limiter without the stub has a recovery time of 16.3 ns, while the one with the parallel resonator is only 11.2 ns. These results demonstrate that the resonator formed by the short-circuited stub effectively accelerates the discharge of accumulated charge in the first-stage SBDs, thereby significantly improving the recovery performance under high-power conditions.

For the transient recovery-time simulation, the peak excitation level was kept within the above-defined model-validity range. The purpose of this transient analysis is not to extrapolate the device behavior beyond its calibrated high-current boundary, but to compare the recovery behavior of different limiter topologies under the same device model and excitation condition. Therefore, the extracted recovery times should be interpreted as circuit-level quantitative results within the established model boundary. Near the upper power boundary, the absolute recovery time may still be influenced by physical effects not fully included in the present equivalent model, such as electrothermal coupling, trapping effects, and local high-field behavior. Nevertheless, the reduction of recovery time from 16.3 ns to 11.2 ns remains meaningful as a topology-dependent comparison because both limiters are evaluated using the same SBD model, excitation waveform, and recovery-time extraction criterion.

### 3.3. Linearity of the Limiter

**Figure 12:**
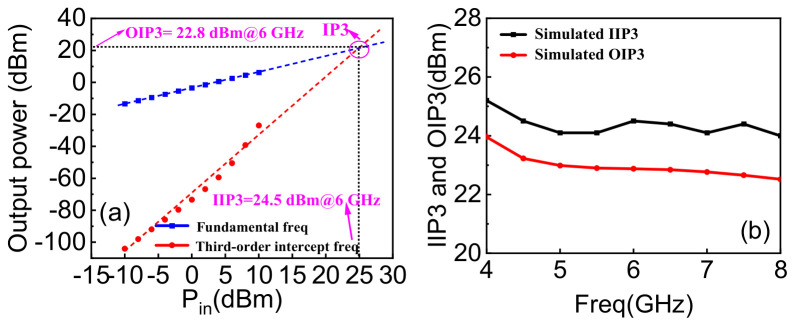
The simulated IP3 of the GaN SBD limiter MMIC: (**a**) IIP3 and OIP3 at 6 GHz. (**b**) OIP3 and IIP3 in the C-band.

Additionally, the third-order intercept point (IP3) is a key indicator for evaluating the linearity of GaN limiters. A two-tone simulation was performed within the operating frequency band to evaluate the input-referred intercept point (IIP3) and output-referred intercept point (OIP3). In Figure 12a, the IP3 value measured at 6 GHz was obtained by analyzing the intersection of the fundamental output power and the third-order intercept frequency. The OIP3 and IIP3 are 22.8 dBm and 24.5 dBm, respectively. Figure 12b shows the OIP3 and IIP3 in the C-band, where OIP3 varies from 22.5 dBm to 23.9 dBm and IIP3 varies between 24.1 dBm and 25.2 dBm.

The recovery characteristics and linearity performance of the proposed limiter are further compared with previously reported microwave limiter designs in Table 3, including key parameters such as recovery time, power capacity, and OIP3. Moreover, unlike several previous works evaluated under low-duty-cycle pulsed excitation, the proposed limiter is characterized under CW excitation, which imposes a more stringent steady-state large-signal stress. Compared with conventional limiter topologies, the proposed parallel-resonator-assisted structure exhibits a shorter recovery time under high-power conditions while maintaining comparable linearity performance in the C-band. The comparison indicates that the introduction of the parallel resonant unit significantly affects the transient recovery behavior of the limiter without causing substantial degradation in linearity characteristics.

## 4. Conclusions

In this work, the recovery characteristics of a high-power GaN Schottky barrier diode (SBD) limiter were investigated using a parallel-resonator-assisted topology and circuit-level transient simulation analysis. An ADS-based transient simulation method combining high-power pulse excitation with a low-power continuous-wave signal was established to analyze the dynamic recovery process from the large-signal limiting state to small-signal steady-state operation. Simulation results show that, under a 50-W input power condition, the introduction of the parallel resonant unit reduces the recovery time from 16.3 ns to 11.2 ns while maintaining favorable linearity characteristics. These results indicate that circuit topology has a significant influence on the recovery behavior of high-power GaN SBD limiters. The proposed simulation approach and topology design provide a useful reference for the circuit-level design and transient analysis of fast-recovery microwave limiters.

## Figures and Tables

**Table 1:** Model Parameters.

Model	Parameters	Values
Intrinsic	*I*_s_ (nA)	0.96
	*R*_S_ (Ω)	1181
	*C*_J,0_ (fF)	0.567
	*N*	1.75
Extrinsic	*C*_pp_ (fF)	0.38
	*C*_pad1_ (fF)	9
	*C*_pad2_ (fF)	16.5
	*C*_pad3_ (fF)	20.5
	*L*_pad_ (pH)	191

**Table 2:** Comparison of circuit parameters for two topologies of the subsequent stage of the limiter.

Topology	Transmission-Line (*TL*_2_)		Parallel Capacitors (*C*_π_ = *C*_D2_/2)	Area of the Limiter (mm^2^)
	Characteristic Impedance	Electrical Length		
Quarter-wavelength topology	*Z*_0_ = 50 Ω	*θ*_2_ = 90° (*λ*/4)	-	3.1 × 1.3
π-type topology	*Z*_2_ = 84 Ω	*θ*_2_ = 45° (*λ*/8)	0.167 pf	2.6 × 1.3

**Table 3:** Comparisons with some of the previous microwave limiter in references.

	Material	Device Type	Freq (GHz)	Size (mm^2^)	OIP3 (dBm)	Recovery Time (ns)	Peak Incident Power (dBm)	Test Condition
[13]	Si	PIN	0.03–0.3	5 × 5	22	-	40	-
[20]	GaAs	PIN	4–8	2.78 × 1.94	20	115	41	CW
							44	PW = 1 μs, DC = 1%
[21]	GaAs	SBD	DC-12	4 × 4	30	-	47	PW = 1 μs, DC = 1%
[5]	GaAs	HEMT + SBD	2.6–3.6	2.5 × 2	-	300	54	PW = 1 ms, DC = 15%
This work	GaN-SiC	SBD	4–8	2.6 × 1.3	22.8	<15	>47	CW

## Data Availability

The original contributions presented in this study are included in the article. Further inquiries can be directed to the corresponding authors.
